# Supplementary material for: Efficient Direct and Limited Environmental Transmission of SARS-CoV-2 Lineage B.1.22 in Domestic Cats
Source: Microbiol Spectr. 2023 May 24;11(3):e02553-22. doi: 10.1128/spectrum.02553-22 (PMC10269887; doi:10.1128/spectrum.02553-22)

Supplemental material

Supplemental Material S1: Activity analysis

Video analysis was performed to investigate the effect of SARS-CoV-2 on activity. A RGB camera was placed in pens A of groups 1, 2, 3, and 4 to record daily sessions of 19 hours, from 3 p.m. to 10 a.m. the next day (1920 x 1080 pixels, 30 FPS). Recordings of DPI -6 to DPI 5 were analyzed, of which 5 recordings were discarded (3 corrupted recordings, one video in which a cat escaped from the pen (before inoculation) and one recording with a flickering light leading to disturbance). Of each recording, the first 2.5 hours and the last 0.5 hour were discarded, because animal caretakers entered the pens during these times. In addition, 2 minutes were discarded around the timestamps that the camera changed from day to night vision (~5 hours into the video) and from night to day vision (~17 hours into the recording).

Activity was quantified using pixel differences. A sampling rate of 1 out of 5 frames was applied, so that 6 frames per second were kept for the analysis. Frames were converted to grayscale and for each pair of subsequent frames, intensity values (ranging from 0 to 255) were compared. A pixel was considered to change when its intensity value differed by more than 40 between subsequent frames (see Figure S1 for an example). To remove extreme outliers caused by light flashes, for example, a moving median of 6 frames was used. Results of the 6 days before inoculation were then compared to those from 5 days after inoculation. Analyses were performed in Python with the OpenCV set of open-source computer vision tools (Bradski, 2000).

Activity results showed a circadian rhythm (Figures S2-S5). Activity was relatively low during the night and peaked around dusk (3-6 hours into each video) and dawn (15-18 hours into each video). This is in line with the crepuscular nature of domestic cats (Cove et al., 2018; Parker et al., 2019).

There was no difference in activity at pen level between the six days before inoculation and 5 days after inoculation (Figure S6). When comparing the 6 days before inoculation with 2 days after inoculation, instead of 5 days after inoculation, there was also no observable difference. The absence of change in activity post inoculation is in line with the absence of clinical symptoms in this study.

References

Bradski, G. (2000). The OpenCV Library. *Dr. Dobb's Journal of Software Tools*.

Cove, M.V., Gardner, B., Simons, T.R. *et al.* Free-ranging domestic cats (*Felis catus*) on public lands: estimating density, activity, and diet in the Florida Keys. *Biol Invasions* **20,** 333–344 (2018).

https://doi.org/10.1007/s10530-017-1534-x

Parker, M., Lamoureux, S., Challet, E. *et al.* Daily rhythms in food intake and locomotor activity in a colony of domestic cats. *Anim Biotelemetry* **7,** 25 (2019). https://doi.org/10.1186/s40317-019-0188-0

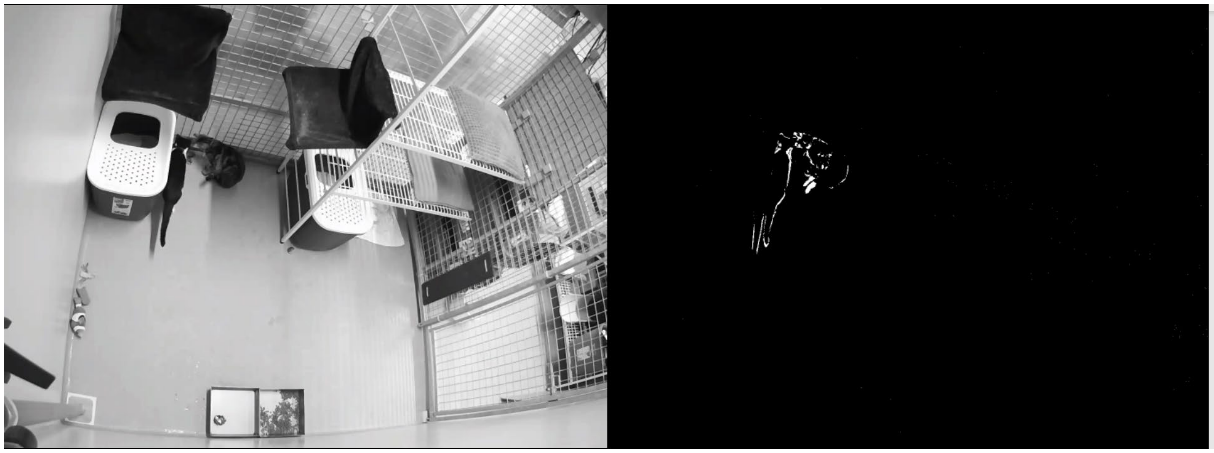

Figure S1. *Quantifying activity with pixel differences. Left: grayscale frame of original video. Right: pixels that changed more than 40 in intensity values between current frame and previous frame shown in white (rest is black).*

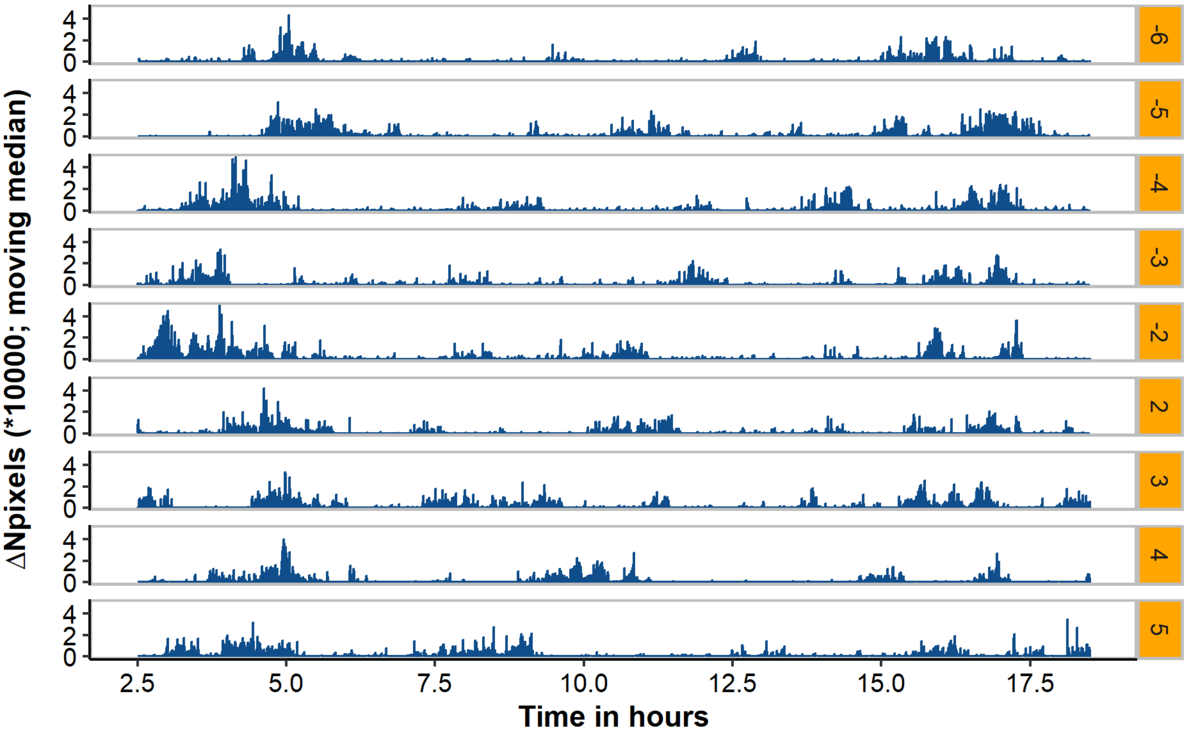

Figure S2. *Moving median of the number of pixels that differed between subsequent frames (ΔNpixels) for the days before and after inoculation for pen 1A. Note: the videos for day -1 and day 1 were unavailable/discarded.*

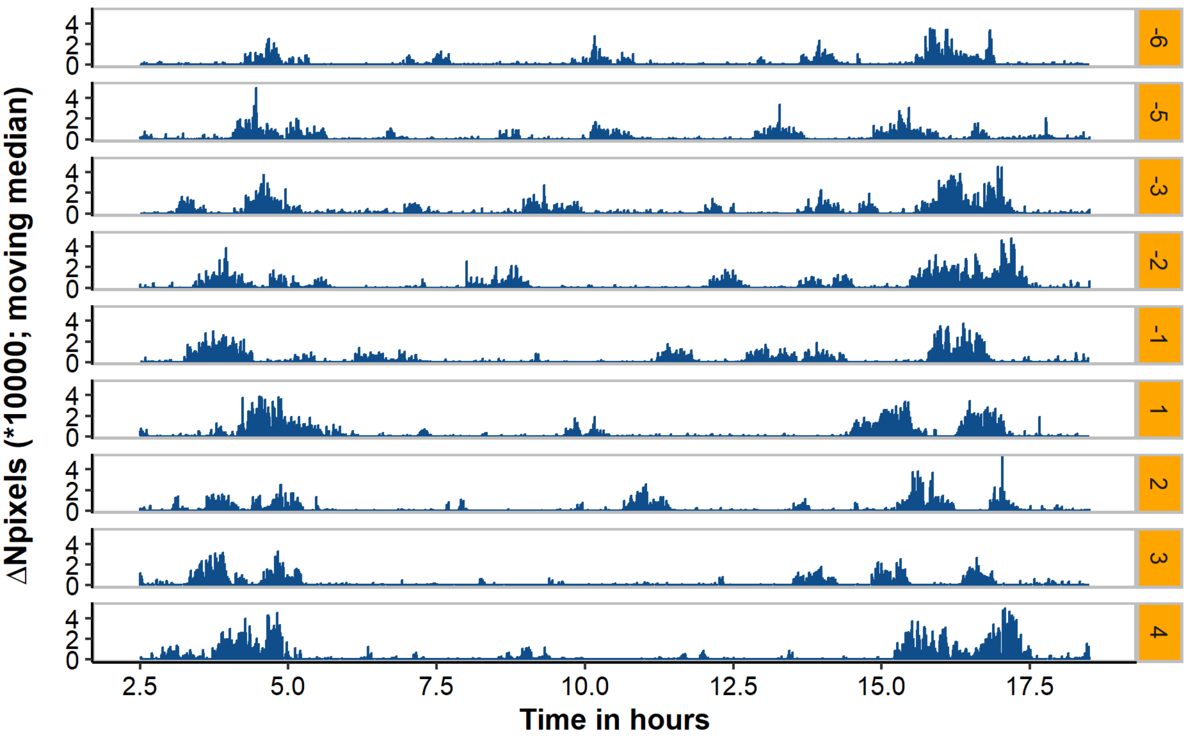

Figure S3. *Moving median of the number of pixels that differed between subsequent frames (ΔNpixels) for the days before and after inoculation for pen 2A. Note: the videos for day -4 and day 5 were unavailable/discarded.*

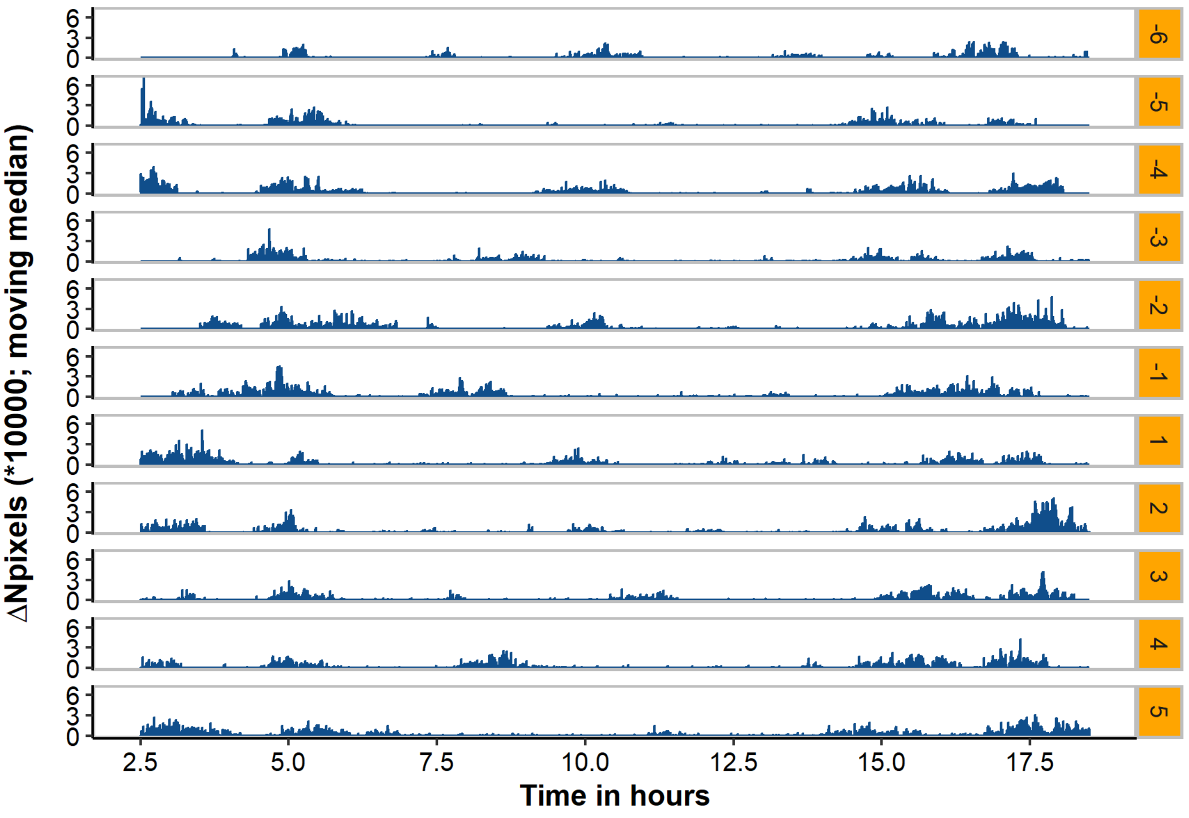

Figure S4. *Moving median of the number of pixels that differed between subsequent frames (ΔNpixels) for the days before and after inoculation for pen 3A.*

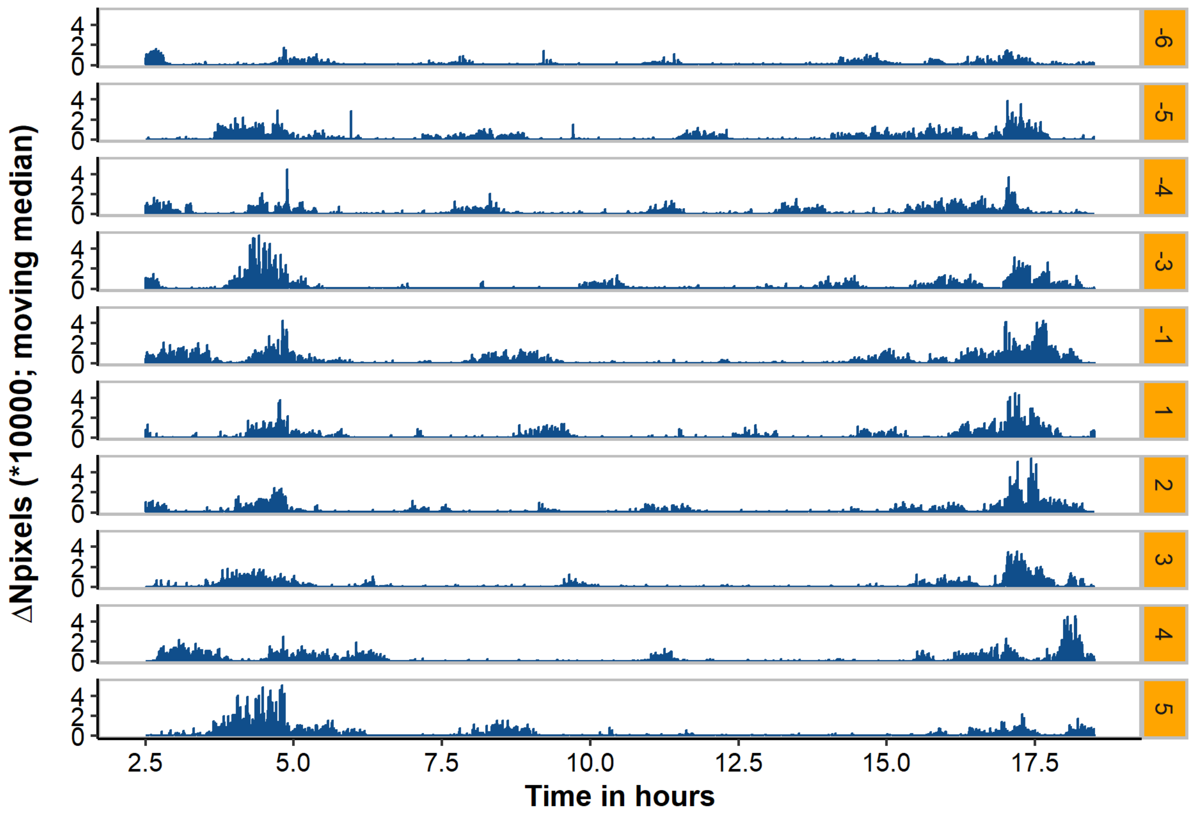

Figure S5. *Moving median of the number of pixels that differed between subsequent frames (ΔNpixels) for the days before and after inoculation for pen 4A. Note: the video for day -2 was unavailable.*

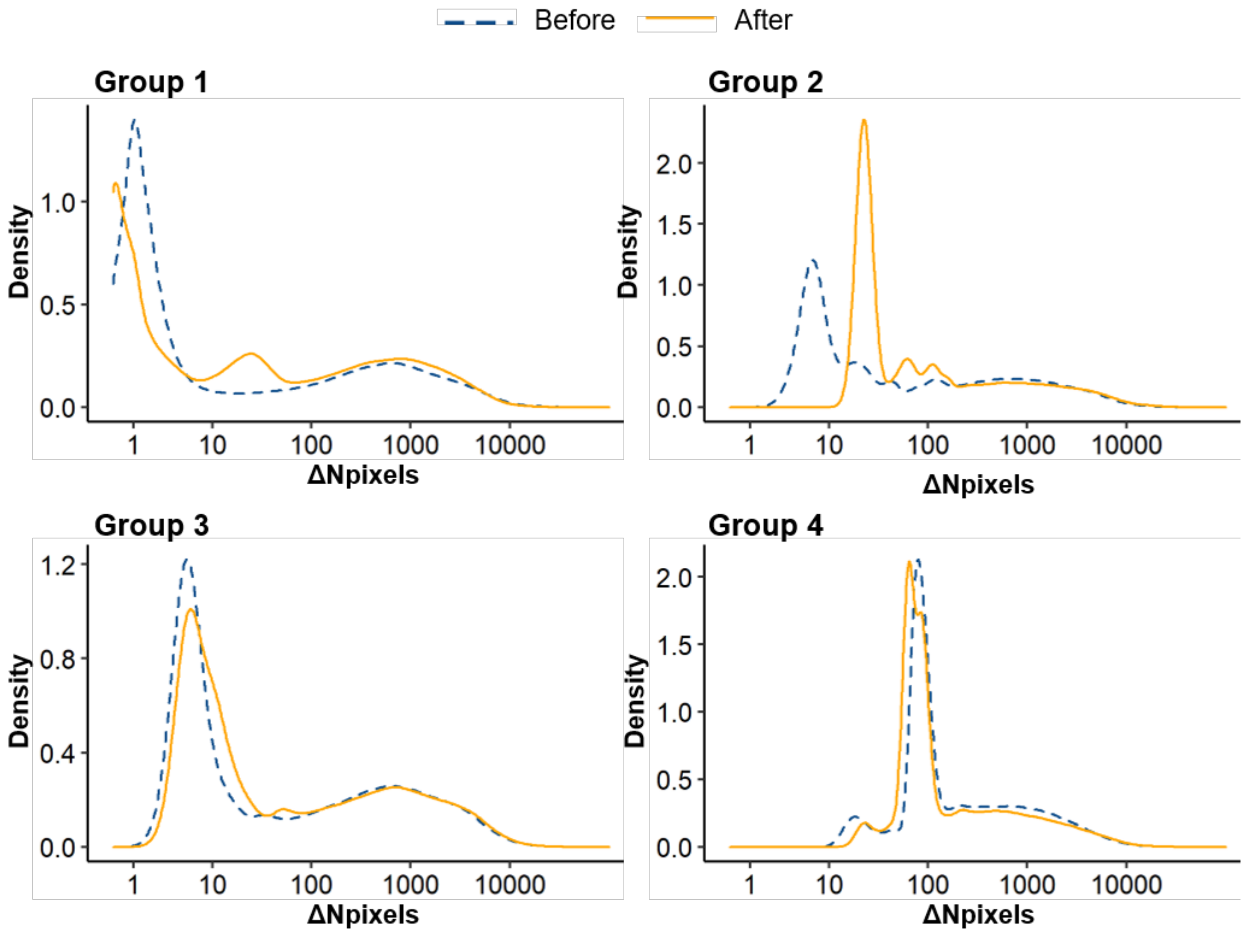

Figure S6. *Density plots of the number of pixels that differed between subsequent frames (ΔNpixels) for the six days before inoculation and five days after inoculation for groups 1, 2, 3, and 4. Note: a (pseudo)* ^{10}*log-scale was used for the x-axis.*

Supplemental material S2: Mathematical analysis

Datasets

Data 1

data1 Criterion Seroconversion only direct contact period

cat ID	start	end	#S	prev	case	history
1.2	1	5	1	0.5	0	{}
1.2	5	6	1	0.5	1	{{0.5,1,5}}
2.2	1	5	1	0.5	0	{}
2.2	5	6	1	0.5	1	{{0.5,1,5}}
3.2	1	6	1	0.5	0	{}
4.2	1	6	1	0.5	0	{}

data2 Criterion seroconversion both direct and indirect period

cat ID	start	end	#S	prev	case	history
1.2	1	5	1	0.5	0	{}
1.2	5	6	1	0.5	1	{{0.5,1,5}}
2.2	1	5	1	0.5	0	{}
2.2	5	6	1	0.5	1	{{0.5,1,5}}
1.3	6	13	1	0	0	{{0.5,1,6}}
1.4	6	23	1	0	0	{{0.5,1,6}}
2.3	6	23	1	0	0	{{0.5,1,6}}
2.4	6	23	1	0	0	{{0.5,1,6}}
3.3	6	23	1	0	0	{{0.5,1,6}}
3.4	6	23	1	0	0	{{0.5,1,6}}
4.3	6	23	1	0	0	{{0.5,1,6}}
4.4	6	23	1	0	0	{{0.5,1,6}}
3.2	1	6	1	0.5	0	{}
4.2	1	6	1	0.5	0	{}

data3 Criterion sgPCR only direct period

cat ID	start	end	#S	prev	case	history
1.2	1	5	1	0.5	0	{}
1.2	5	6	1	0.5	1	{{0.5,1,5}}
2.2	1	5	1	0.5	0	{}
2.2	5	6	1	0.5	1	{{0.5,1,5}}
3.2	1	6	1	0.5	0	{}
4.2	1	2	1	0.5	1	{}

data4 Criterion sgPCR both direct and indirect period

cat ID	start	end	#S	prev	case	history
1.2	1	5	1	0.5	0	{}
1.2	5	6	1	0.5	1	{{0.5,1,5}}
2.2	1	5	1	0.5	0	{}
2.2	5	6	1	0.5	1	{{0.5,1,5}}
4.2	1	2	1	0.5	1	{}
1.3	6	7	1	0	1	{{0.5,1,6},{0.5,6,6}}
1.4	6	7	1	0	0	{{0.5,1,6},{0.5,6,6}}
1.4	7	13	1	0.5	0	{{0.5,1,6},{0.5,6,6}}
1.4	13	23	1	0	0	{{0.5,1,6},{0.5,6,6},{0.5,7,13}}
2.3	6	23	1	0	0	{{0.5,1,6},{0.5,6,6}}
2.4	6	23	1	0	0	{{0.5,1,6},{0.5,6,6}}
3.3	6	23	1	0	0	{{0.5,1,6}}
3.4	6	23	1	0	0	{{0.5,1,6}}
4.3	6	23	1	0	0	{{0.5,1,6},{0.5,2,6}}
4.4	6	23	1	0	0	{{0.5,1,6},{0.5,2,6}}
3.2	1	6	1	0.5	0	{}

Algorithm

Definitions

```
Clear[HazardEt, IntegratEt, E0, R0, totalE, Exposure,
 datalinelikelihood, logdatalikelihood, profilelikelihoodβ,
 profilelikelihoodμ, profilelikelihoodR, profileβ, profileμ, profileR]

(* environmental contamination D[Env[t],t] ==
 ϕ(μ) It - μ E(t) with ϕ(μ) standardised *)

In[24]:= (* instantaneous hazard rate when It is constant
 in the interval and the amount is E0 at the start*)
```

In[25]:= HazardEt[t_, It_, E0_, μ_] :=

$$\frac{\mu\left(1 - e^{-\mu t}\right)}{-1 + e^{-\mu} + \mu}\,\text{It} + e^{-\mu t}\,\text{E0}$$

```
(* accumulated over time period when It is constant during
  the interval and E0 is the amount at the start of the interval
  Note: the standardisation makes it equal to It for
        t=1 and E0=0 *)
```

In[27]:= IntegratEt[t_, It_, E0_, μ_] :=

$$\frac{-1 + e^{-\mu t} + \mu t}{-1 + e^{-\mu} + \mu}\,\text{It} + \frac{1 - e^{-\mu t}}{\mu}\,\text{E0}$$

```
(* amount already present at the start of this interval
 given historical exposure(s) with shedding from infected (I/N),
value in history⟦x,1⟧, during previous intervals ending with history⟦x,3⟧
 and starting at history⟦x,2⟧ time units and decay then continues
 until the start of the interval under consideration at t0. *)
```

In[29]:= E0[history_, μ_, t0_] := If[Length[history] ≤ 0, 0,

$$\sum_{x=1}^{\text{Length[history]}} (\text{If}[t0 \geq \text{history}⟦x, 3⟧,\ \text{HazardEt}[\text{history}⟦x, 3⟧ - \text{history}⟦x, 2⟧,\ \text{history}⟦x, 1⟧, 0, \mu] * \text{Exp}[-\mu\,(t0 - \text{history}⟦x, 3⟧)], 0])$$

]

```
In[ ]:= totalE[μ_, It_] := μ/(-1 + e^-μ + μ) It

In[ ]:= R0[β_, μ_, T_] := β totalE[μ, 1] T

In[ ]:= Exposure[n_, μ_, data_] :=
         IntegratEt[data[[n, 3]] - data[[n, 2]], data[[n, 5]], E0[data[[n , 7]], μ, data[[n, 2]]], μ]

In[ ]:= (* dataline likelihood with I/N data[[n,6]] during the interval
         (length data[[n,7]]) present resulting in data[[n,5]] positive individuals
         and data[[n,4]] infection negative individuals. Exposure history is
         in data[[n,8]] with three values per entry (see explanantion of E0) *)

In[ ]:= datalinelikelihood[n_, β_, μ_, data_] :=
         If[Exposure[n, μ, data] > 10^-10, ((1 - Exp[-β Exposure[n, μ, data]])^data[[n,6]]
            Exp[-β Exposure[n, μ, data]]^If[data[[n,6]]>0,0,1]), (1;
           If[data[[n, 6]] > 0, Print[" Dataline ", n, " not possible"]])]


In[ ]:= (* for likelihood optimisation we add the
         Log of all the observed independent likelihoods *)

In[ ]:= logdatalikelihood[β_, μ_, data_] := Sum[Log[datalinelikelihood[n, β, μ, data]], {n, 1, Length[data]}]

In[ ]:= (* for profile likelihood we calculate the
         parameter β given the value of μ or vice versa *)

In[ ]:= profileμ[μ_, data_] :=
         β /. Flatten[FindMaximum[{logdatalikelihood[β, μ, data], β > 0}, {β, 0.1}]][[2]]

In[ ]:= profileβ[β_, data_] :=
         μ /. Flatten[FindMaximum[{logdatalikelihood[β, μ, data], μ > 0}, {μ, 0.1}]][[2]]

In[ ]:= profileR[R_, T_, data_] := {R / (totalE[μ, 1] T), μ} /. Flatten[
            FindMaximum[{logdatalikelihood[R / (totalE[μ, 1] T), μ, data], μ > 0}, {μ, 0.1}]][[2]]

In[ ]:= (* these are then the profilelikelihoods *)

In[ ]:= profilelikelihoodμ[μ_, data_] := logdatalikelihood[profileμ[μ, data], μ, data]

In[ ]:= profilelikelihoodβ[β_, data_] := logdatalikelihood[β, profileβ[β, data], data];

In[ ]:= profilelikelihoodR[R_, T_, data_] :=
          logdatalikelihood[profileR[R, T, data][[1]], profileR[R, T, data][[2]], data];
```

Profile likelihoods final model

The profile likelihood of the decay rate parameter μ, estimated value 2.73 day^{-1} (0.77 - 15.82)

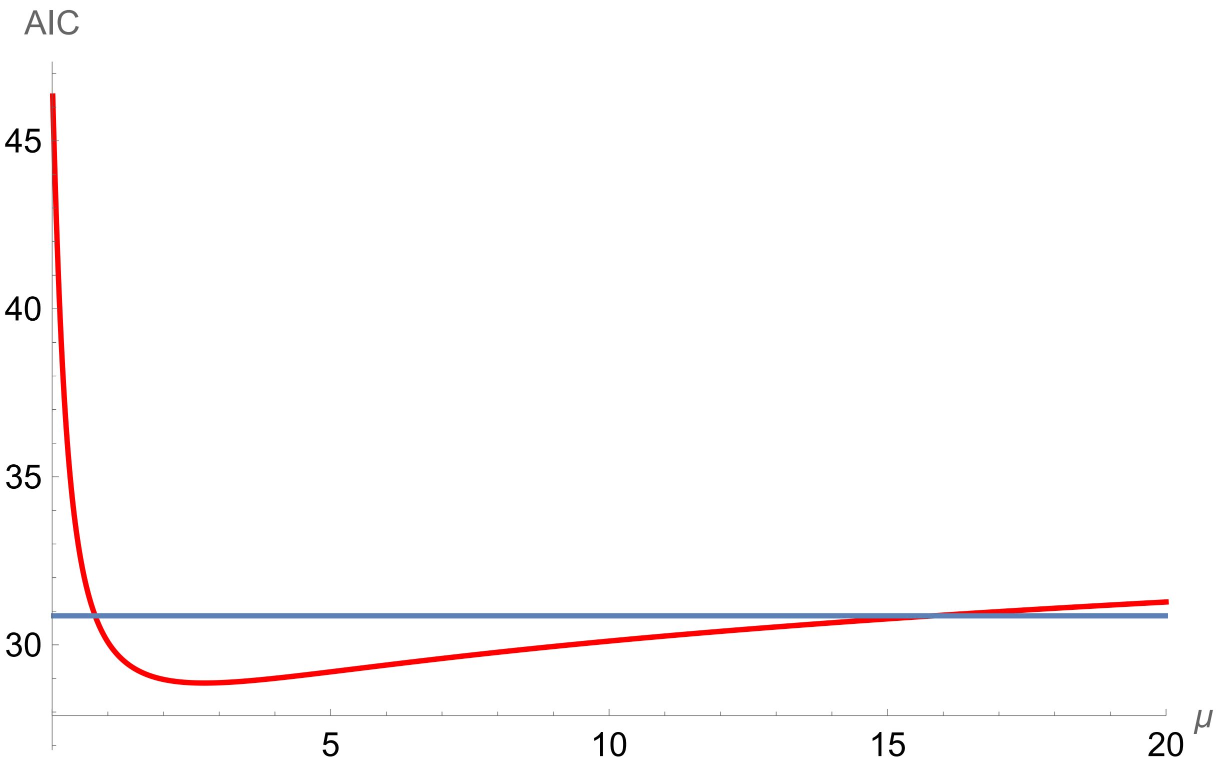

The profile likelihood of the transmission rate parameter β, estimated value 0.23 day^{-1} (0.06 - 0.54).

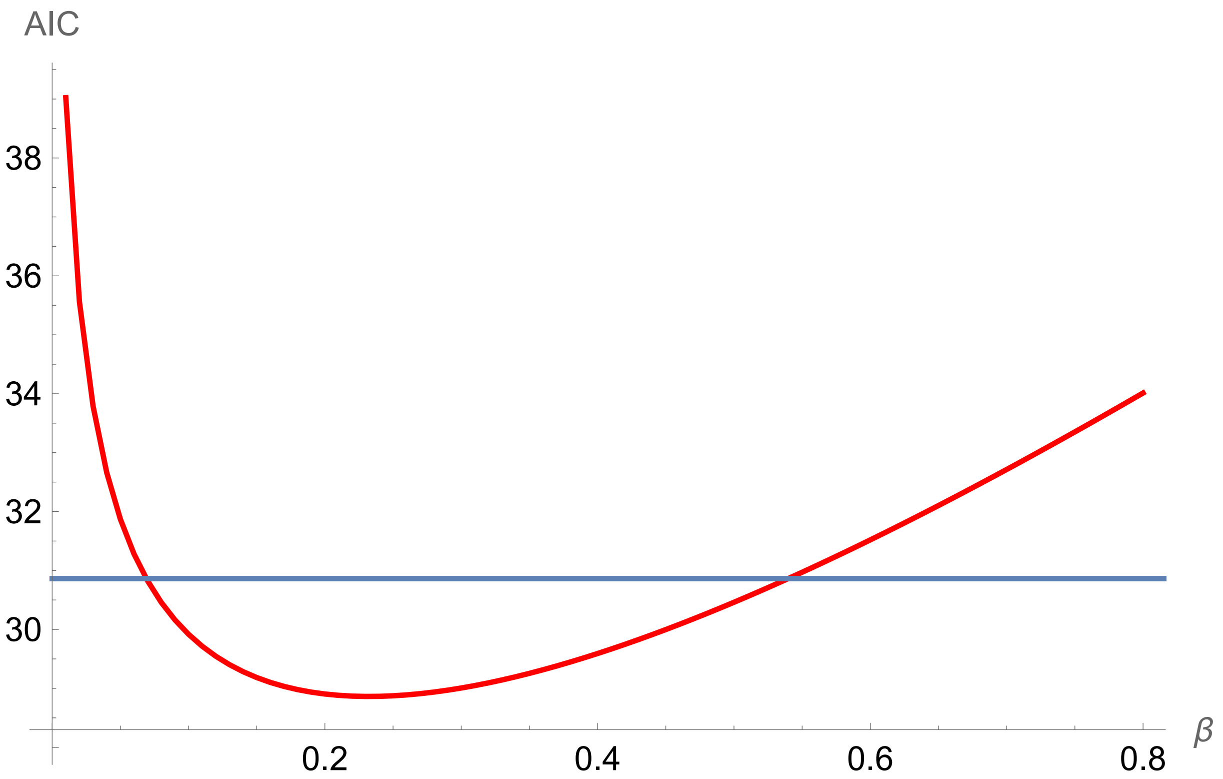

The profile likelihood of the reproduction ratio R, estimated value 2.12 (0.92 - 4.08).

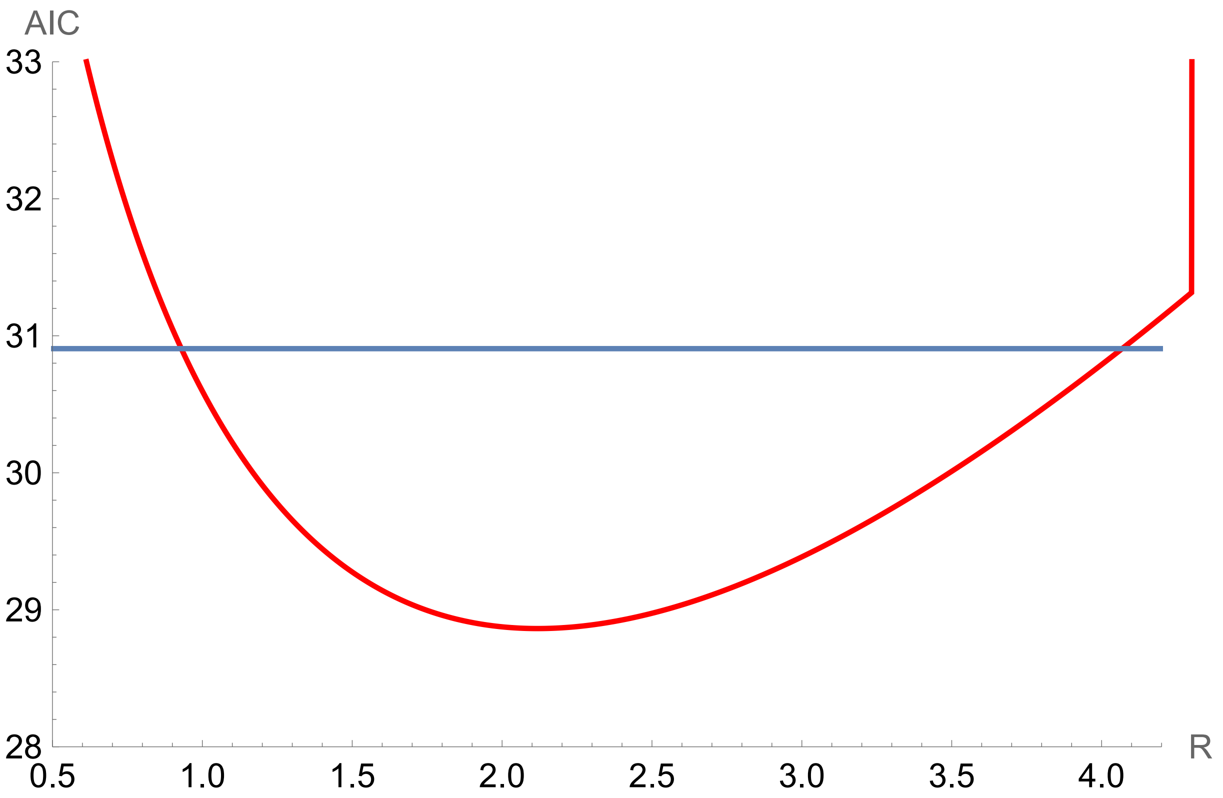

Supplement: Supplemental file 1 — Supplemental material. Download spectrum.02553-22-s0001.pdf, PDF file, 0.8 MB [file spectrum.02553-22-s0001.pdf]
